# Delirium and IL-6 added to clinical scores improves their performance: a prospective analysis of CALL, PREDI-CO, MRS score applied to a population of patients admitted to internal medicine ward

**DOI:** 10.1007/s11739-023-03336-2

**Published:** 2023-06-17

**Authors:** Vieri Vannucchi, Lorenzo Pelagatti, Fabio Barone, Laura Bertini, Tommaso Celli, Nunzia Boccia, Francesca Veneziani, Barbara Cimolato, Giancarlo Landini

**Affiliations:** 1 Internal Medicine, Santa, Maria Nuova Hospital, Piazza di Santa Maria Nuova 1, 50121 Florence, Italy; 2 High-Dependency Unit, AOU Careggi, Florence, Italy; 3 School of Medicine, University of Florence, Florence, Italy; 4 Laboratory of Clinical Pathology, Santa Maria Nuova Hospital, Florence, Italy; 5 Laboratory of Clinical Pathology, San Giovanni di Dio Hospital, Florence, Italy

**Keywords:** Covid-19, CALL, PREDI-CO, MRS, Score, Outcome, Delirium,il-6

## Abstract

This study aimed to evaluate the effectiveness of various scoring systems in predicting in-hospital mortality for COVID-19 patients admitted to the internal medicine ward. We conducted a prospective collection of clinical data from patients admitted to the Internal Medicine Unit at Santa Maria Nuova Hospital in Florence, Italy, with confirmed pneumonia caused by SARS-CoV-2. We calculated three scoring systems: the CALL score, the PREDI-CO score, and the COVID-19 in-hospital Mortality Risk Score (COVID-19 MRS). The primary endpoint was in-hospital mortality. : A total of 681 patients were enrolled in the study, with a mean age of 68.8 ± 16.1 years, and 54.8% of them were male. Non-survivors had significantly higher scores in all prognostic systems compared to survivors (MRS: 13 [12- 15] vs. 10 [8-12]; CALL: 12 [10-12] vs. 9 [7-11]; PREDI-CO: 4 [3-6] vs. 2 [1-4]; all p<0.001). The receiver operating characteristic (ROC) analysis yielded the following area under the curve (AUC) values: MRS 0.85, CALL 0.78, PREDI-CO 0.77. The addition of Delirium and IL6 to the scoring systems improved their discriminative ability, resulting in AUC values of 0.92 for MRS, 0.87 for CALL, and 0.84 for PREDI-CO. The mortality rate increased significantly across increasing quartiles (p<0.001). In conclusion the COVID-19 in-hospital Mortality Risk Score (MRS) demonstrated reasonable prognostic stratification for patients admitted to the internal medicine ward with SARS-CoV-2-induced pneumonia. The inclusion of Delirium and IL6 as additional prognostic indicators in the scoring systems enhanced their predictive performance, specifically in determining in-hospital mortality among COVID-19 patients.

## Introduction

In December 2019, a new coronavirus family member named Severe Acute Respiratory Syndrome Coronavirus 2 (SARS-CoV-2) was discovered in Wuhan, Hubei, China. Over the following 2 years, it rapidly spread worldwide, leading to a pandemic.

COVID-19 exhibits a wide range of signs and symptoms, varying from asymptomatic cases to severe pneumonia. Approximately 20% of patients experience acute respiratory distress syndrome (ARDS) and multi-organ failure (MOF) [1, 2], resulting in high morbidity and mortality rates.

Internal medicine wards have played a crucial role in managing patients with COVID-19. In Italy, 75–90% of COVID-19 patients required hospitalization in a medical setting. These patients often had complex medical conditions and comorbidities, necessitating internal medical management in addition to treating the infectious aspect of the disease.

In hospitalized patients, SARS-CoV-2 infection can progress rapidly, even in those with mild symptoms. Consequently, it is vital to identify individuals at risk of developing ARDS and severe forms of the disease. Bacterial pneumonia has long been a leading cause of hospitalization, particularly among older patients and those with pre-existing medical conditions [3]. To address this, prognostic and clinical scores have been developed over time to predict the mortality risk for patients with bacterial pneumonia [4–6]. These scores help identify patients at a higher risk of complications and severe disease, enabling appropriate therapeutic and care decisions.

To stratify patients with COVID-19, specific scores have been developed for assessing prognosis in the emergency department and for hospitalized patients. However, the efficacy of these scores varies [7]. Most of the existing studies validating these scores have been conducted on small population samples, with specific ethnic or demographic characteristics, making generalizability to different contexts challenging [8–10]. Limited data are available in the literature regarding the validation and performance of clinical scores in patients with COVID-19 admitted to internal medicine wards.

We conducted an analysis of three scores for patients with COVID-19: the CALL score [8], the PREDI-CO score [9], and the COVID-19 in-hospital Mortality Risk Score (COVID-19 MRS) [10]. These scores were selected based on their relevance in assessing COVID-19 patients, their accuracy and reliability in predicting patient outcomes, and their widespread adoption in the medical community.

The CALL score has demonstrated its ability to accurately predict mortality risk in COVID-19 patients. Similarly, the PREDI-CO score and COVID-19 MRS were specifically developed for COVID-19 patients and consider a range of clinical and laboratory parameters to forecast disease severity and outcomes. We chose these clinical scores not only for their effectiveness but also for their simplicity, as they incorporate clinical and laboratory parameters commonly utilized in clinical practice and routinely monitored in all COVID-19 patients.

The objective of our study was to assess and compare the prognostic performance of these different scores in predicting in-hospital mortality among patients admitted to the internal medicine ward.

## Materials and methods

### Study design

We conducted a single-center prospective observational study involving consecutively hospitalized patients with SARS-CoV-2 pneumonia in Santa Maria Nuova Hospital in Florence, Italy, specifically in the Internal Medicine Unit, which has a total of 56 beds. The study was conducted between March 13, 2020, and May 30, 2021. The study protocol received approval from the local hospital ethics committee.

Patients were included in the study if they met the following criteria upon admission: age equal to or above 18 years, evidence of COVID-19 confirmed by a positive polymerase chain reaction on a nasopharyngeal swab, and admission to the internal medicine ward.

The primary objective of this study is to prospectively validate the clinical scores known as CALL, PREDI-CO, and COVID-19 in-hospital Mortality Risk Score. We aim to assess their predictive capability regarding in-hospital mortality in a real-life population of patients admitted to the internal medicine department and diagnosed with COVID-19. The secondary objective of the study is to evaluate the potential statistical association between IL-6 levels and delirium with higher hospital mortality: If such an association is found, we will explore whether incorporating IL-6 levels and delirium into the clinical scores improves their performance.

The measurement of IL-6 was performed on all patients upon admission to the internal medicine ward as a routine practice. The inclusion of this parameter and its evaluation in clinical scores was based on the well-established significance of IL-6 as a prognostic factor, which has been extensively documented in the literature. Elevated levels of IL-6 have shown a progressive correlation with disease severity [11].

Additionally, all patients were systematically assessed for delirium upon admission and throughout their entire hospital stay. A clear association between COVID-19 and delirium has been observed. The existing literature indicates a higher incidence of delirium among patients with COVID-19, particularly those in intensive and sub-intensive care settings. Moreover, patients with delirium have exhibited increased mortality rates and longer hospital stays [12].

### Patients

The patients were enrolled upon admission to the internal medicine ward, and written and/or verbal consent for the processing of personal data was obtained from each patient in accordance with the Ethical Committee. Clinical data were extracted from the patients' medical records, including blood tests and blood gases obtained upon admission. Both clinical and laboratory data were collected from electronic medical records and the clinical analysis laboratory database.

Delirium development was monitored in each patient upon arrival and during their stay in the ward. The CALL score, PREDI-CO score, and COVID-19 in-hospital Mortality Risk Score (COVID-19 MRS) were calculated for each patient. Only in-hospital mortality and complications were considered in the analysis.

The study design, data collection, data analysis, and manuscript preparation were performed by the authors, who ensure the accuracy and completeness of the data. The corresponding authors were responsible for writing the submitted manuscript. Data collection was conducted within the COMETA project, which received approval from the local ethics committee (CEAVC 18436). The project was funded by the Tuscany region, without any sponsor-funded editorial support.

### Scores

- The CALL Score, developed by Ji Dong et al. [8], is a predictive tool designed to assess the risk of severe disease progression in hospitalized patients with COVID-19 pneumonia. Its purpose is to stratify patients based on the potential severity of the disease, facilitating prompt allocation of appropriate care settings and treatments. The authors identified independent risk factors for disease progression and incorporated these factors into the CALL Score: Comorbidity, age, lymphocyte count, and LDH levels (Table 1). This scoring system ranges from a minimum of 4 points to a maximum of 13, with a higher score indicating an elevated risk of disease progression.
- The receiver operating characteristic (ROC) curve analysis demonstrated an area under the curve (AUC) of 0.91 (95% CI 0.86–0.94).
- The COVID-19 in-hospital Mortality Risk Score (COVID-19 MRS) is a clinical score developed by Fumagalli et al. [10]. It is designed to predict in-hospital mortality and is based on clinical and laboratory parameters that are easily identifiable at the time of hospital admission. To create the score, the variables identified through multivariate analysis were divided into tertiles, and each tertile was assigned a score ranging from 1 to 3. These scores were then used to generate a final score (Table 1), with a minimum score of 6 and a maximum score of 18.
- The performance of the COVID-19 MRS ROC curve demonstrated an Area Under the Curve (AUC) of 0.90 (95% CI 0.87–0.93).
- The PREDI-CO Score originates from a retrospective multicenter cohort study conducted by Bartoletti et al. [9]. The primary objective of this study was to identify independent risk factors associated with the progression of respiratory failure to a severe stage.

**Table 1:** The set of scores investigated, CALL score, PREDI-CO Score, COVID-19 in-hospital Mortality Risk Score (COVID-19 MRS) [8–10] with information about the type of parameter, cut-off and points assigned for each score

COVID-19 in-hospital Mortality Risk Score (COVID-19 MRS)			CALL score		
Parameter	Cut-off	Points	Parameter	Cut-off	Points
Age	< 62	1	Comorbidity^b^	No	1
	62 – 74	2		Yes	4
	≥ 75	3	Age	≤ 60	1
Comorbidity^a^	≤ 1	1		> 60	4
	2 – 3	2	Lymphocyte (10^9^/L)	> 1	1
	≥ 4	3		≤ 1	3
Respiratory rate (breaths per minute)	≤ 20	1	LDH (U/L)	≤ 250	1
	21 – 24	2		250–500	2
	≥ 25	3		> 500	3
PaO_2_/FiO_2_	≥ 300	1	PREDI-CO score		
	299 – 236	2	Parameter	Cut-off	Points
	≤ 235	3	Age	≥ 70 years	1
Creatinine (mg/dL)	≤ 0.82	1	Obesity	BMI > 30/m2	1
	0.83 – 1.12	2	Temperature (°C)	≥ 38	1
	≥ 1.13	3	Respiratory rate	≥ 22/min	1
Platelet count (10^9^/L)	≥ 212	1	Creatinine (mg/dL)	≥ 1	1
	156 – 211	2	CRP (mg/dL)	≥ 10	1
	≤ 155	3	LDH (IU/L)	≥ 350	1
			Lymphocytes (10^9^/L)	≥ 900	1

^a^Hypertension, diabetes mellitus, cardiovascular disease, previous stroke/TIA, CODP, cancer, depression, dementia

^b^Hypertension, diabetes, cardiovascular disease, liver disease, asthma, chronic lung disease, HIV infections, and malignancy for at least 6 months

The multivariate analysis revealed several independent risk factors associated with the development of respiratory failure. These factors include: age ≥ 70 years, obesity (BMI > 30 kg/m^2^), admission temperature ≥ 38°C, respiratory rate ≥ 22 breaths per minute, creatinine ≥ 1 mg/dL, C-reactive protein (CRP) ≥ 10 mg/dL, lactate dehydrogenase (LDH) ≥ 350 IU/L, and lymphocytes ≤ 900 per mm^3^.

To create the scoring system (Table 1), each parameter was assigned a score. In this scoring system, a score of 1 was given to each parameter, except for C-reactive protein, which was assigned a score of 2. Consequently, each patient received a score ranging from 0 to 9 points based on these parameters.

The ROC curve analysis demonstrated an area under the curve (AUC) of 0.89 (95% CI: 0.86 - 0.92) for the PREDI-CO Score.

### Statistical analysis

Continuous variables were expressed as mean and standard deviation while categorical ones as proportion and percentage. In general, the t-Student test was used for the comparison of normally distributed continuous variables and the χ^2^ analysis for categorical variables. The Mann–Whitney U test was used to evaluate the difference in the scoring systems between survivor and non-survior. The odds ratio (OR) values and the 95% confidence intervals were calculated using a univariate and multivariate logistic regression analysis. For the multivariate analysis, variables with p < 0.1 in the univariate analysis were selected. All two-sided p-values < 0.05 (95% CI) were considered statistically significant. The effectiveness of the scores was carried out through the analysis of the receiver operating characteristic (ROC) curves and the calculation of the respective area under the curve (AUC) values. Multivariate analysis showed that IL-6 and delirium are independent risk factors associated with respiratory failure and increased in-hospital mortality. Therefore, we recalculated the CALL, PREDI-CO and MRS scores with the addition of the delirium and IL-6 variables. The analysis was performed using the Statistical Package for Social Sciences 21 (SPSS Inc. Chicago IL. USA). The graphs were made with GraphPad Prism 9 (GraphPad Software, LLC).

## Results

### Patients

The study comprised a total of 681 patients, with an average age of 68.8 ± 16.1 years. The mortality rate was 12.3% (84 deceased patients). The mean duration of hospital stay in the ward was 9.4 ± 8.8 days. Table 2 presents the additional clinical characteristics of the overall population. Furthermore, Table 3 provides information on the laboratory parameters and vital signs of our population upon admission to the hospital.

**Table 2:** Clinical characteristics of the patients examined during the study: statistical parameters on age, sex and hospitalization days of all patients, of patients deceased in hospital stay, of survivors, and relative p-values

Clinical characteristic	Total population n = 681	Mortality during in hospital stay		p Value
		Non-survivors n = 84 (12.3%)	survivors n = 597 (87.3%)	
Mean age ± standard deviation	69.8 ± 16.1	84.5 ± 8.3	67.7 ± 15.9	0.01
Mean age (median, interquartile range)	71 (59.5–83.0)	86.5 (79.2–90.0)	69.0 (58.0–80.0)	0.01
Age ≥ 70 years (percentage)	375 (55.1)	81 (96.4)	294 (49.2)	0.001
Male sex (percentage)	379 (55.4)	46 (54.8)	333 (55.8)	0.9
Mean days in hospital stay ± standard deviation	9.4 ± 8.8	9.2 ± 7.3	9.4 ± 9.0	0.9

Comorbidities	Total population n = 681 (%)	Mortality during in hospital stay		p Value
		Non-survivors n = 84 (12.3%)	Survivors n = 597 (87,3%)	
Active Smoker	102 (15)	12 (14.3)	90 (15.1)	1
≥ One comorbidity	471 (69.2)	80 (95.2)	391 (65.5)	0.001
≥ Three comorbidity	220 (32.3)	61 (72.6)	159 (26.6)	0.001
Arterial hypertension	360 (52.9)	64 (76.2)	296 (49.6)	0.001
Diabetes mellitus	135 (19.8)	23 (27.4)	112 (18.8)	0.08
Ischemic heart disease	60 (8.8)	17 (20.2)	43 (7.2)	0.001
Heart failure	25 (3.7)	12 (14.3)	13 (2.2)	0.001
Ischemic or haemorrhagic stroke/TIA	23 (3.4)	4 (4.8)	19 (3.2)	0.5
Hypertensive heart disease	20 (2.9)	3 (3.6)	17 (2.8)	0.7
Atrial fibrillation	67 (9.8)	18 (21.4)	49 (8.2)	0.001
Chronic lung disease^a^	100 (14.7)	18 (21.4)	82 (13.7)	0.07
Chronic renal failure	94 (13.8)	34 (40.5)	60 (10.1)	0.001
Active cancer	44 (6.5)	11 (13.1)	33 (5.5)	0.02
Chronic Inflammatory diseases^b^	16 (2.3)	0 (0)	16 (2.7)	0.2
Dementia	121 (17.8)	45 (53.6)	76 (12.7)	0.001
Liver disease	6 (0.9)	2 (2.4)	4 (0.7)	0.2
Obesity (BMI > 30)	68 (10)	6 (7.1)	62 (10.4)	0.4
HIV	2 (0.3)	0 (0)	2 (0.3)	1

Therapies and complications during hospitalization	Total population n = 681 (%)	Mortality during in hospital stay		p Value
		Non-survivors n = 84 (12.3%)	Survivors n = 597 (87.3%)	
Oxygen therapy	642 (94.3)	81 (97.6)	561 (94.1)	0.3
Non invasive ventilation (NIV)	312 (45.8)	51 (60.7)	261 (43.7)	0.005
No NIV	369 (54.2)	33 (39.3)	336 (56.3)	0.1
Dexamethasone	538 (79)	65 (77.4)	473 (79.6)	0.7
EBPM/Fondaparinux	631 (92.7)	69 (83.1)	562 (94.3)	0.001
Antibiotic therapy	513 (75.3)	70 (84.3)	443 (74.3)	0.06
Remdesivir	184 (27)	3 (3.6)	181 (30.4)	0.001
Tocilizumab	35 (5.1)	2 (2.4)	33 (5.5)	0.3
Bilateral pulmonary infiltrates on CT	252 (37)	22 (95.7)	230 (92)	1
ICU admission	42 (6.2)			
Delirium	61 (9)	30 (35.7)	31 (5.2)	0.001
Acute coronary syndrome	11 (1.6)	4 (4.8)	7 (1.2)	0.04
Ischemic stroke	3 (0.4)	1 (1.2)	2 (0.3)	0.3

Comorbidities and therapies of the patients examined during the study: total number of patients, of deceased, survivors and relative p-values for each specific parameter

^a^Chronic lung diseases: includes chronic obstructive pulmonary disease, moderate to severe asthma, pulmonary fibrosis

^b^Chronic inflammatory diseases: chronic inflammatory bowel diseases (Crohn’s disease and ulcerative colitis), rheumatic diseases (such as rheumatoid arthritis, SLE) and finally psoriatic diseases, such as psoriasis and psoriatic arthritis

**Table 3:** Laboratory parameters and vital parameters at the time of admission to the hospital: mean and standard deviation calculated on the number of all patients, on the deceased and on the survivors for each parameter and relative p values

Laboratory exam	Population n = 681	Mortality during in hospital stay		p Value
		Non-survivors n = 84 (12.3%)	Survivors n = 597 (87.3%)	
	Mean ± Standard deviation	Mean ± Standard deviation	Mean ± Standard deviation	
Haemoglobin (g/dL)	13.0 ± 1.9	12.3 ± 2.2	13.1 ± 1.8	0.002
Platelet (10^9^/L)	223.4 ± 92.9	195.4 ± 86.6	227.3 ± 93.1	0.003
White cells (10^9^/L)	7.9 ± 4.3	10.4 ± 7.0	7.5 ± 3.6	0.001
Neutrophils (10^9^/L)	6.3 ± 4.5	9.0 ± 5.9	5.9 ± 4.2	0.001
Lymphocytes (10^9^/L)	1.1 ± 1.05	1.2 ± 2.8	1.1 ± 0.6	0.7
D-Dimer (µg/mL)	1612 ± 3673	2533 ± 3080	1501 ± 3725	0.03
Fibrinogen (mg/dL)	641.9 ± 101.8	599.0 ± 131.0	646.0 ± 97.0	0.002
Creatinine (mg/dL)	1.1 ± 1.3	2.0 ± 2.5	1.0 ± 0.9	0.001
AST (UI/L)	46.9 ± 65.0	57.0 ± 124.0	45.0 ± 51.0	0.1
ALT (UI/L)	41.2 ± 59.0	44.0 ± 121.0	40.0 ± 44	0.6
LDH (UI/L)	315.0 ± 140.0	400.0 ± 175.0	303.0 ± 130.0	0.001
Total bilirubin (mg/dL)	0.8 ± 3.9	0.6 ± 0.3	0.8 ± 4.1	0.7
C reactive protein (mg/dL)	8.3 ± 8.6	13.2 ± 17.0	7.6 ± 6.4	0.001
Procalcitonin (ng/mL)	1.1 ± 4.9	3.0 ± 6.5	0.8 ± 4.6	0.004
IL-6 (pg/mL)	58.5 ± 137.6	106.0 ± 135.0	52.0 ± 136.0	0.009
High sensitivity troponin I (ng/L)	83.4 ± 731.4	483.0 ± 2130.0	33.0 ± 152.0	0.001
BNP (pg/mL)	206.1 ± 377.2	490.0 ± 623.0	166.0 ± 309.0	0.001
Vital parameters				
Body temperature (°C)	36.7 ± 0.9	36.4 ± 0.6	36.7 ± 1.0	0.001
Systolic blood pressure (mmHg)	133 ± 20	124 ± 21	134 ± 20	0.001
Diastolic blood pressure (mmHg)	76 ± 11	71 ± 12	77 ± 11	0.001
Heart rate (bpm)	85 ± 16	83 ± 17	85 ± 15	0.3
Respiratory rate (acts/min)	19 ± 4	20 ± 5	19 ± 3	0.001
PaO_2_/FiO_2_	272.5 ± 97.8	203.0 ± 97.0	282.0 ± 93.0	0.001

Among the population, 69.2% had at least one comorbidity. The most prevalent comorbidities were arterial hypertension (52.9%), diabetes mellitus (19.8%), and cognitive impairment (17.8%). In the subgroup of deceased patients, almost all individuals (95.2%) had at least one comorbidity, while 72.6% had three or more. Non-invasive ventilation was administered to almost half of the patients (45.8%), with 169 (54.2%) receiving C-PAP mode, 46 (14.7%) receiving Bi-PAP mode, and 97 (31.1%) receiving both modes.

One notable complication observed during hospitalization was the occurrence of delirium, affecting 61 patients (9% of the population). Within this specific group, mortality rate was approximately 50% (30 deaths versus 31 recoveries).

### Scores

**Fig. 1:**
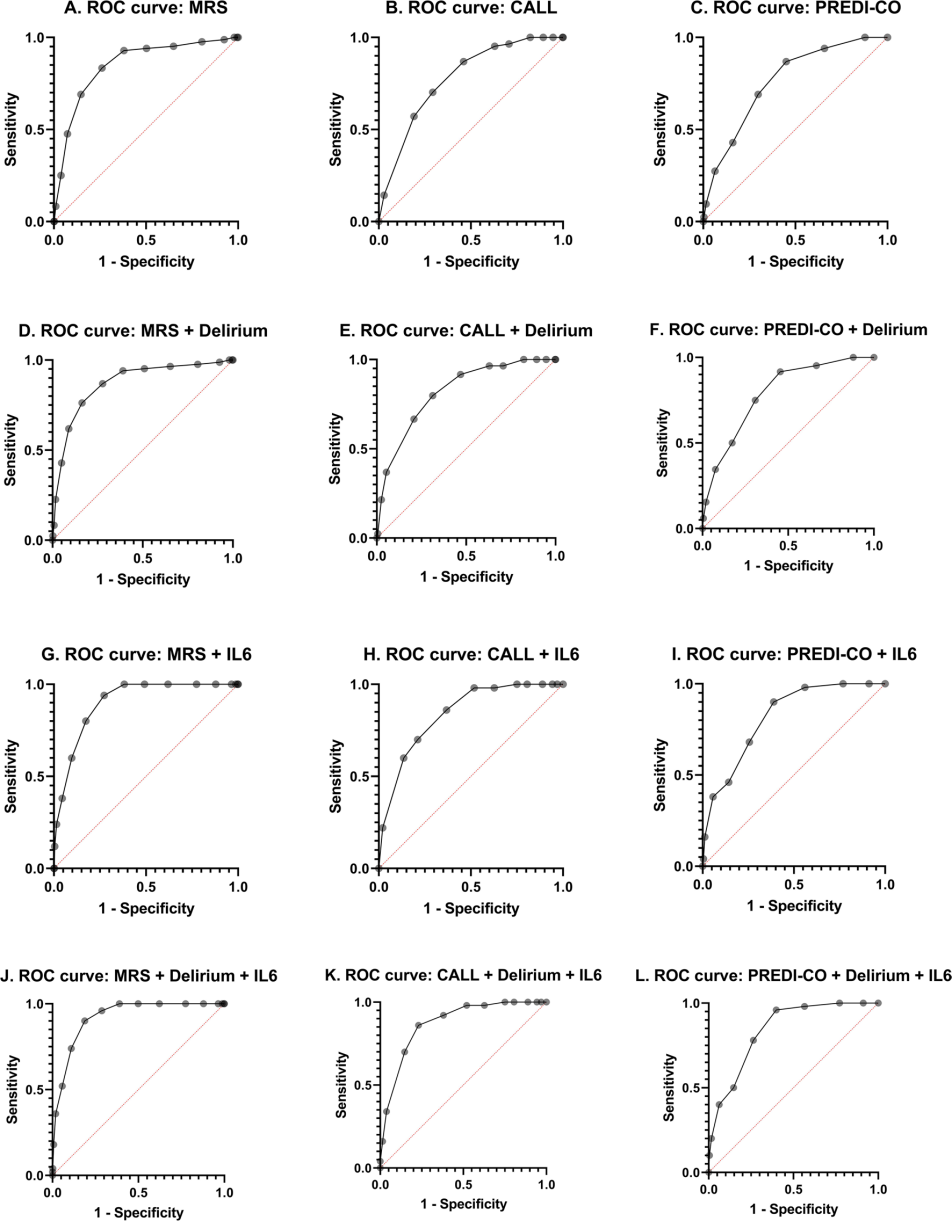
Cartesian diagrams of the ROC curves for the three scores under consideration (from the left to the right: MRS score, CALL score, PREDI-CO score; from the top to the bottom: ROC curves for each score calculated alone, with the addition of a single variable and the addition of the two variables examined

All the scores were significantly higher in non-survivors (n = 84) compared to survivors (n = 597): CALL score 12 [10–12] vs. 9 [7–11], p < 0.001, PREDI-CO score 4 [3–6] vs. 2 [1–4], p < 0.001, MRS score 13 [12–15] vs. 10 [8–12], p < 0.001. To assess the prognostic stratification ability, ROC curve analysis was conducted. The results showed that the MRS score had the highest predictive ability for in-hospital mortality (0.85, 95% CI 0.81–0.89), followed by the CALL score (0.78, 95% CI 0.73–0.82) and the PREDI-CO score (0.77, 95% CI 0.71–0.81) (Fig. 1a–c).

Furthermore, a multivariate analysis was performed to determine independent risk factors associated with respiratory failure and increased in-hospital mortality. The analysis revealed that IL-6 and delirium were independent risk factors (p < 0.001)

We performed recalculations of the CALL, PREDI-CO, and MRS scores by incorporating the delirium variable. The delirium variable was assigned a score of 3 for the CALL score, 1 point if absent, 3 for the MRS score (1 if absent), and 1 for the PREDI-CO score (0 if absent) based on the OR values from our dataset.

The scores, when calculated with the addition of delirium, demonstrated improved performance compared to the original scores (see Fig. 1d–f): MRS score AUC 0.87 (95% CI 0.83–0.91), CALL score AUC 0.82 (95% CI 0.77–0.86), PREDI-CO score AUC 0.79 (95% CI 0.74–0.84).

IL-6 levels were divided into tertiles, and a different score was assigned to each tertile. For the MRS score, 1 point was assigned for IL-6 < 17.1, 2 for 17.1 ≤ IL-6 ≤ 48.8, and 3 for IL-6 > 48.8. Each tertile with a higher IL-6 value was associated with a statistically significant increase in mortality compared to the previous tertile (p < 0.001). Regarding the CALL score, a score of 1 was assigned to values lower than 17.1, while a score of 3 was assigned for values ≥ 17.1. In the first group, the percentage of deaths was 1.9% compared to 15% in the second group (p < 0.001). Lastly, for the PREDI-CO score, values lower than 17.1 were assigned a score of 0, while values ≥ 17.1 were assigned a score of 1. In the first group, the percentage of deaths was 1.9% compared to 15% in the second group (p < 0.001).

Upon recalculation of the scores with the inclusion of IL-6, the following results were obtained (Fig. 1g–i): MRS score AUC 0.90 (95% CI 0.87–0.93), CALL score AUC 0.84 (95% CI 0.79–0.89), PREDI-CO score AUC 0.82 (95% CI 0.77–0.87).

By incorporating IL-6 and delirium into the scores, the highest level of performance was achieved. The scoring system for IL-6 and delirium remains as described earlier. The results are as follows (Fig. 1j–l): MRS score AUC 0.92 (95% CI 0.89–0.95), CALL score AUC 0.87 (95% CI 0.83–0.92), PREDI-CO score AUC 0.84 (95% CI 0.79–0.89).

**Fig. 2:**
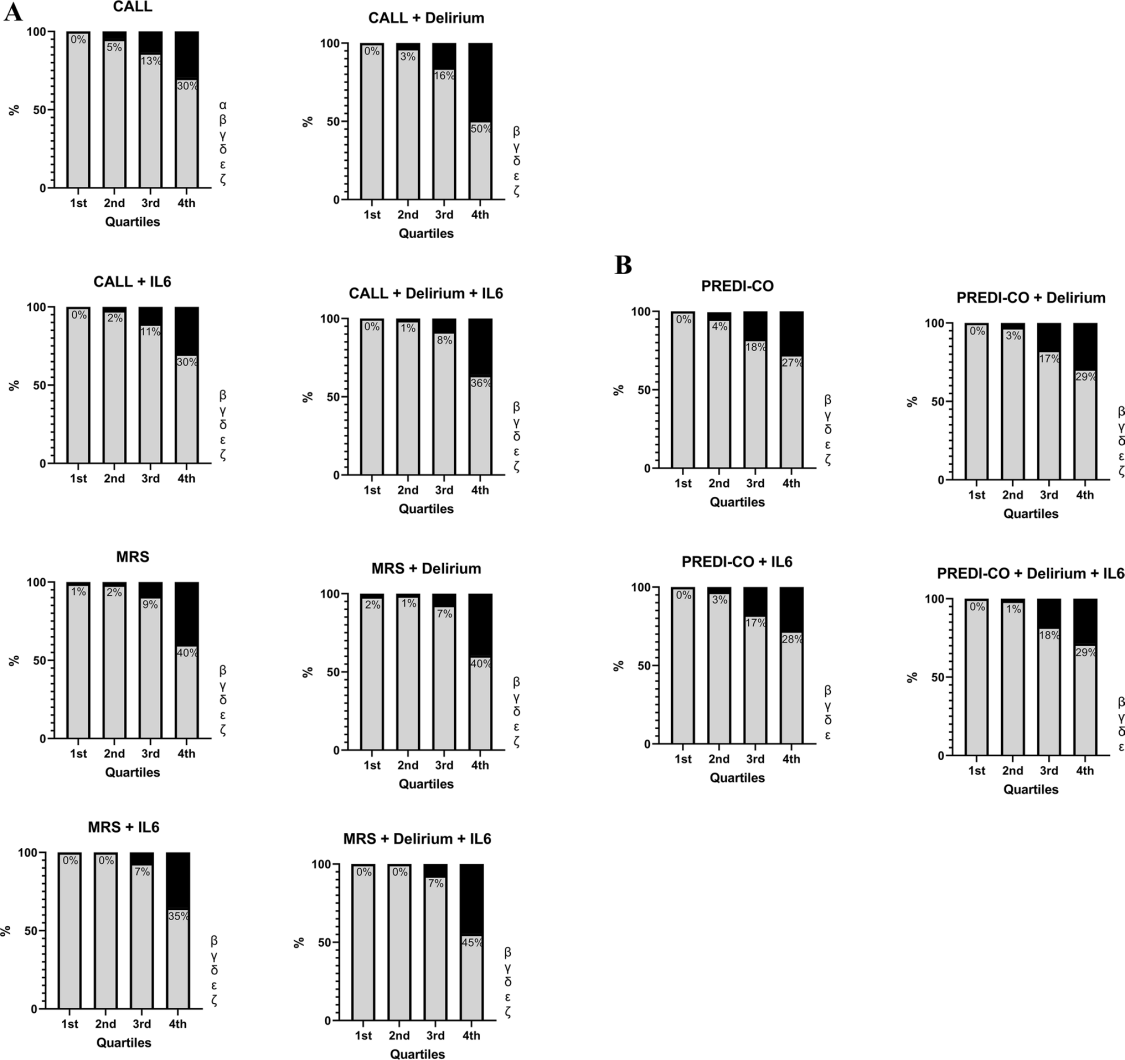
**A** Histograms of CALL score and MRS score showing the mortality rate in each quartile calculated alone, with the addition of a single variable and the addition of the two variables examined (color code: Grey=Survivor; Black=Non survivor). α: p<0.05 1st-2nd quartile; β: p<0.05 1st-3rd quartile; γ: p<0.05 1st-4th quartile; δ: p<0.05 2nd-3rd quartile; ε: p<0.05 2nd-4th quartile; ζ: p<0.05 3rd-4th quartile. **B** Histograms of PREDI-CO score showing the mortality rate in each quartilecalculated alone, with the addition of a single variable and the addition of the two variables examined (color code: Grey=Survivor; Black=Non survivor). α: p<0.05 1st-2nd quartile; β: p<0.05 1st-3rd quartile; γ: p<0.05 1st-4th quartile; δ: p<0.05 2nd-3rd quartile; ε: p<0.05 2nd-4th quartile; ζ: p<0.05 3rd-4th quartile

Finally, we assessed the mortality rate across different quartiles of the scores (refer to Table 4; Fig 2a, b). The prevalence of in-hospital mortality significantly increased from the first to the fourth quartile of the 4C mortality score, with minimal presence in the first quartile and disproportionately high rates in the fourth quartile.

**Table 4:** Scores values in the whole population and based on in-hospital mortality

In-hospital mortality				
	All patients (n = 681)	Survivors (n = 597)	Non-survivors (n = 84)	p
CALL	10 [7–11]	9 [7–11]	12 [10–12]	0.001
MRS	10 [8–12]	10 [8–12]	13 [12–15]	0.001
PREDI-CO	3 [1–4]	2 [1–4]	4 [3–6]	0.001
CALL + delirium	11 [8–13]	10 [8–13]	13 [12–14]	0.001
MRS + delirium	11 [9–13]	11 [9–13]	15 [14–16]	0.001
PREDI-CO + delirium	3 [1–4]	2 [1–4]	4 [3–6]	0.001
CALL + IL6	12 [10–14]	12 [9–14]	16 [13–16]	0.001
MRS + IL6	12 [10–14]	11 [10–14]	16 [15–17]	0.001
PREDI-CO + IL6	3 [2–5]	3 [2–5]	5 [4–7]	0.001
CALL + delirium + IL6	13 [11–15]	13 [10–14]	16 [15–17]	0.001
MRS + delirium + IL6	13 [11–16]	12 [11–15]	18 [16–19]	0.001
PREDI-CO + delirium + IL6	3 [2–5]	3 [2–5]	6 [5–7]	0.001

## Discussion

This study demonstrates that risk scores exhibit good performance in predicting in-hospital mortality among COVID-19 patients. Notably, the mortality rate significantly increases across quartiles, with the first quartile showing a negligible mortality rate of less than or equal to 1%, indicating a very low-risk category. The escalating mortality in higher quartiles assists clinicians in prognostic stratification. The novelty of this study lies in the evaluation of these scores within an internal medicine setting, as no such studies exist in the literature. Utilizing clinical scores for COVID-19 enables early prognosis stratification of patients admitted to internal medicine wards.

We specifically chose to analyze the performance of the CALL Score due to its easily obtainable items at admission (age and comorbidities) and laboratory values commonly used in clinical practice [8]. The score was previously validated in a Chinese population of 208 patients [8]. Although our study group demonstrated good predictive performance for mortality, the area under the curve (AUC) was lower than that reported in the original article (AUC 0.91, 95% CI 0.86–0.94). The differences between their results and ours could be attributed to variations in population demographics, a higher mean age (69.8 ± 16.1 vs. 44.0 ± 16.3 years), treatment protocols, and predominant severe acute respiratory syndrome coronavirus 2 (SARS-CoV-2) subtypes [13]. In a smaller Italian population admitted to internal medicine wards (n = 210), Grifoni et al. obtained similar results to our study (AUC 0.768, 95% CI 0.705–0.823) [14]. Similarly, Innocenti et al. [7] analyzed the score’s performance in a large population of patients admitted to a University Hospital, reporting slightly lower performance than ours (AUC 0.74, 95% CI 0.70–0.78).

On the other hand, this study represents the initial attempt at external validation of the PREDI-CO score and COVID-19 in-hospital Mortality Risk Score (COVID-19 MRS). The MRS score was validated in a small Italian geriatric population of 221 consecutive patients with COVID-19 aged ≥ 75 years admitted to two hospitals [10]. Our results showed a comparable performance to that of the authors (AUC 0.85 vs. 0.87). The PREDI-Co score, developed to predict severe respiratory failure (SRF) among hospitalized COVID-19 patients, was evaluated for its applicability as a mortality predictor in our ward. Although the score demonstrated good performance, our result is worse than the one obtained by Bartoletti et al. [9].

Cognitive impairment was frequently observed in our patient sample, with an overall prevalence of 18%. It ranked as the fourth most common comorbidity after hypertension, diabetes mellitus, and cardiovascular disease. This observation aligns with a study conducted on 1970 patients hospitalized for COVID-19 pneumonia in a Spanish tertiary hospital [15]. Regarding mortality, our data reveals that dementia was present in over 50% of deceased patients and exhibited a strong association with in-hospital mortality (OR 6.0; 95% CI 2.7–13.2). A recent meta-analysis involving 24 studies and over 40,000 patients also highlights the close relationship between cognitive impairment and poor prognosis [16].

Cognitive impairment primarily affects the elderly population, who often possess intrinsic physiological frailty and numerous comorbidities that independently contribute to the risk of death. Dementia is frequently associated with the presence of the apolipoprotein E4 gene, which is widely expressed in lung tissue and may predispose individuals to an exaggerated inflammatory response, potentially facilitating the onset of acute respiratory distress syndrome (ARDS) [17]. Several analyses of large datasets suggest a possible correlation between the presence of the ApoE4 allele and an increased risk of mortality in COVID-19 patients. However, it is important to note that the correlation between ApoE4, dementia, and COVID-19 mortality remains not fully understood, requiring further research for confirmation [18–21].

In our patient sample, elevated levels of interleukin 6 (IL-6) measured upon hospital admission were associated with an increased risk of death. These findings align with existing literature. Grifoni et al. [11] and a meta-analysis by Zhu et al. [22] also confirm the close association between in-hospital mortality and elevated IL-6 levels. In our population, 90% (76 out of 84) of deceased patients exhibited increased IL-6 values (using a reference cutoff of 27 pg/mL), providing high sensitivity in predicting in-hospital mortality. Conversely, 97% of patients who recovered had levels below 27 pg/mL. The inclusion of IL-6 as a parameter has improved the performance of clinical scores.

Delirium emerged as the most frequent complication in our population, affecting 61 patients (approximately 10% of the population). Two different meta-analyses by Shao et al. [23] and Pranata et al. [24] demonstrate a significantly higher occurrence of delirium in hospitalized COVID-19 patients, estimated at 32% and 27%, respectively. However, our lower percentage may be due to potential underestimation, as delirium onset was not evaluated during the long-term hospitalization period after discharge. Delirium is a common complication in hospitalized patients, particularly in the elderly population, with approximately 10–15% of hospitalized patients developing this syndrome [25]. COVID-19 patients appear to have a higher incidence of delirium due to potential direct involvement of the central nervous system, systemic mechanisms such as hypoxemia and oxidative stress resulting from lung damage, and the manifestation of encephalopathy secondary to pro-inflammatory cytokine hyperproduction [26]. Additionally, environmental factors accompanying hospital stays may predispose patients to this complication [27]. Regarding mortality, approximately 50% of patients who develop delirium during hospitalization do not survive, consistent with the findings of the meta-analysis by Shao et al. [23]. Delirium, already associated with a twofold increase in the risk of death, particularly in the elderly population [28], serves as an excellent predictor of in-hospital mortality in COVID-19 patients.

Based on these results, the addition of delirium as an additional evaluation factor improves the performance of each prognostic score. While IL-6 augmentation enhances the predictive power of clinical scores, delirium alone enhances the performance of COVID-19 clinical scores and may offer a faster and more cost-effective approach compared to measuring IL-6 levels. In conclusion, incorporating delirium into COVID-19 clinical scores enhances their performance, and although IL-6 remains a valuable predictor, delirium alone may offer a more practical and cost-effective approach. Continuous research and evaluation of different predictors are crucial for developing accurate and efficient clinical scoring systems for predicting outcomes in COVID-19 patients.

## Limitations

The single-center design of the present study is a noteworthy limitation. Consequently, the generalizability of these results may be compromised due to variations in local admission and management policies. The implementation of a standardized data collection template restricted the potential for subjective data interpretation. Moreover, the observational nature of our analysis prevents us from drawing definitive conclusions regarding the clinical factors influencing mortality and their associations with therapeutic strategies, which were also subject to adaptations over time.

## Conclusions

Prognostic scores serve as valuable tools for stratifying the prognosis of patients with COVID-19 who are admitted to internal medicine wards. These scores have been examined and have demonstrated a strong predictive capability for hospitalized patients. Additionally, it has been observed that delirium acts as a predictor of in-hospital mortality, specifically in elderly patients with COVID-19 pneumonia. The incorporation of delirium into the existing three prognostic scores has been found to enhance their prognostic value.

Scores, as zero-cost tools, play a crucial role in our day-to-day lives by aiding in the effective stratification of our patients. It is imperative that we make use of these valuable tools to enhance our understanding of patient outcomes and guide appropriate interventions.

## Data Availability

The datasets generated during and/or analysed during the current study are available from the corresponding author on reasonable request.
